# Feasibility of Provider-Initiated HIV Testing and Counselling of Tuberculosis Patients Under the TB Control Programme in Two Districts of South India

**DOI:** 10.1371/journal.pone.0007899

**Published:** 2009-11-19

**Authors:** Sophia Vijay, Soumya Swaminathan, Preetish Vaidyanathan, Aleyamma Thomas, L. S. Chauhan, Prahlad Kumar, Sonali Chiddarwar, Beena Thomas, Puneet K. Dewan

**Affiliations:** 1 National Tuberculosis Institute, Bangalore, India; 2 Tuberculosis Research Centre, Chennai, India; 3 Central Tuberculosis Division, Directorate General of Health Services, Ministry of Health and Family Welfare, New Delhi, India; 4 MHI for mother and child, Lucknow, India; 5 World Health Organization, Regional Office for Southeast Asia, New Delhi, India; McGill University Health Center, Montreal Chest Institute, Canada

## Abstract

**Background:**

Provider-initiated HIV testing and counselling (PITC) is internationally recommended for tuberculosis (TB) patients, but the feasibility, effectiveness, and impact of this policy on the TB programme in India are unknown. We evaluated PITC of TB patients across two districts in India considered to have generalized HIV epidemics, Tiruchirappalli (population 2.5 million) and Mysore (population 2.8 million).

**Methodology/Principal Findings:**

Starting June 2007, healthcare providers in both districts were instructed to ascertain HIV status for all TB patients, and refer those with unknown HIV status to the nearest Integrated Counselling and Testing Centre (ICTC)—often in the same facility—for counselling and voluntary HIV testing. All TB patients registered from June 2007 to March 2008 were followed prospectively. Field investigators assessed PITC practices and abstracted data from routine TB programme records and HIV counselling registers to determine the proportion of TB patients appropriately evaluated for HIV infection. Patient records were traced to determine the efficiency of referral links to HIV care and antiretroviral treatment (ART). Between July 2007 and March 2008, 5299 TB patients were registered in both study districts. Of the 4701 with unknown HIV status at the time of TB treatment initiation, 3368 (72%) were referred to an ICTC, and 3111 (66%) were newly tested for HIV. PITC implementation resulted in the ascertainment of HIV status for 3709/5299 (70%) of TB patients, and detected 200 cases with previously undiagnosed HIV infection. Overall, 468 (8.8%) of all registered TB patients were HIV-infected; 177 (37%) were documented to have also received any ART.

**Conclusions:**

With implementation of PITC in India, HIV status was successfully ascertained for 70% of TB patients. Previously undiagnosed HIV-infection was detected in 6.4% of those TB patients newly tested, enabling referral for life-saving anti-retroviral treatment. ART uptake, however, was poor, suggesting that PITC implementation should include measures to strengthen and support ART referral, evaluation, and initiation.

## Introduction

The HIV/AIDS epidemic has increased the global tuberculosis (TB) burden, and has focused attention on the necessity to closely coordinate TB and HIV/AIDS control programme services [1]. Early detection of HIV infection among TB patients offers the opportunity to promptly link patients to HIV care interventions, such as cotrimoxazole prophylactic treatment (CPT) and antiretroviral treatment, which can reduce suffering and death [2].

In India, among the estimated 1.852 million tuberculosis cases occurring annually, an estimated 5.2%—more than 96,000 persons— are HIV-infected [3]. Surveillance has shown that, in places where HIV seroprevalence is high, HIV infection among TB patients is common [4]. Access to HIV counselling, testing, care and treatment services—including free antiretroviral treatment (ART)—is rapidly expanding under the National AIDS Control Programme (NACP) [5].

As of 2007, the policy of the Government of India's Revised National Tuberculosis Control Programme (RNTCP) and the National AIDS Control Organization (NACO) was to offer referral for HIV testing only to those patients with behavioural risk factors for HIV infection or evidence of opportunistic infections [6]. This approach proved to be operationally challenging, as routine ascertainment of HIV behavioural risk factors in patients or their partners was not always possible due to crowded outpatient clinical services.

The World Health Organization (WHO) and Joint United Nations programme on HIV-AIDS (UNAIDS) now recommend Provider Initiated HIV Testing and Counselling (PITC) for all TB patients in all settings [7]. PITC guidelines hold health-care providers responsible for proactively offering HIV testing and counselling to all persons with any sign or symptom of HIV infection or associated opportunistic infections, including tuberculosis.

A study from South India has shown that a majority of TB patients expressed their willingness to accept HIV testing [8], but implementation of the PITC policy in the programme has never been tested in India. To guide TB-HIV policy in India, we prospectively evaluated the implementation of PITC in the programme for all tuberculosis patients registered in two districts in south India. The study objectives were to evaluate the feasibility and effectiveness of PITC in detecting HIV-infection in TB patients and to assess the efficiency of linkage to HIV care and treatment for HIV infected TB patients.

## Methods

### Setting

The study was conducted in two districts: Mysore (population 2.8 million) in Karnataka state and Tiruchirappalli (population 2.5 million) in Tamil Nadu state. These districts have been classified as high priority for HIV interventions by NACO on the basis of consistently having >1% HIV seroprevalence during sentinel surveillance at antenatal clinics [9]. In the two districts, RNTCP services are available through a network of primary health care facilities which provide general health services, including TB diagnostic and treatment services. Tuberculosis services are decentralized, with quality-assured smear microscopy and directly observed treatment through all public health facilities and through a network of community DOT providers. All TB patients initiated on treatment were registered at the six sub district level TB programme management units in each district. Voluntary HIV counselling and testing services were offered through a NACP-supported network of 57 integrated counselling and testing centres (ICTC): 27 in Mysore and 30 in Tiruchirappalli. Free antiretroviral treatment (ART) was provided at two ART centres (1 in each district), where HIV-infected patients are screened for ART eligibility and offered HIV treatment and care. These service delivery sites under NACP follow the national guidelines for counselling, testing, care and treatment of HIV-infected patients [10].

### Design and Definitions

A prospective observational study was undertaken after introducing the PITC referral procedure for all the TB patients registered under RNTCP from 1 July, 2007 to 31 March 2008 in two districts of South India. The outcomes assessed were feasibility of referral for HIV testing, its effectiveness in terms of ascertainment of HIV status for TB patients and the initiation of ART in HIV-infected TB patients. Known HIV status was defined as HIV test result from an ICTC prior to TB diagnosis. A TB patient was considered to be referred to an ICTC or ART centre if the referral was documented on the TB treatment card, a referral form, or the relevant register in the ICTC or ART centre. A patient was presumed to have reached the ICTC or ART centre if evidence of their visit was recorded in the corresponding registers in the ICTC or ART centre.

### PITC Implementation

Physicians and health staff were directed to assess all TB patients for HIV status. If the patient's HIV status was known, that information was to be recorded on the modified treatment card. If the HIV status was unknown, physicians were asked to advise the patients to undergo voluntary HIV testing as soon as possible at the ICTC in the same facility (if available) or at the nearest ICTC. HIV testing was performed as per NACP guidelines which includes verbal consent, pre- and post-test counselling for all clients [10]. HIV test results were communicated by the counsellors to the referring physician through patients and directly, provided patients did not object to ensure timely care and support to HIV infected patients.

Physicians were asked to refer HIV-infected TB patients to the ART centre as soon as possible, after at least 2 weeks of anti-TB treatment in order to reduce the risk of transmission to others. Those patients already known to be HIV infected prior to TB treatment were also referred to ART centres for modification of their ART regimen. National ART guidelines recommend that CD4 cell testing be performed at the initial evaluation in all clients, and recommend ART for patients with pulmonary tuberculosis having a CD4 count <350/mm^3^ or all patients with any extrapulmonary TB disease —who would be classified as WHO clinical stage 4—regardless of CD4 count [11]. ART is to be initiated as soon as possible if the CD4 count is <200/mm^3^, or soon after completion of the intensive phase of anti-TB treatment if CD4 count was 200–350/mm^3^. ART centres were asked to send TB patients back to the referring physician with feedback on the initiation of ART.

To initiate implementation of PITC for TB patients, along with district health authorities we held training sessions in the districts for all public sector physicians, ICTC counsellors, NGO and private sector partners participating in the programme, TB programme staff, and ART centre staff. Training consisted of PITC and ART referral procedures, recording requirements, and the introduction and distribution of standardized referral forms for ICTC and ART.

### Data Collection and Analysis

Information was prospectively collected on all TB patients registered from 1 July 2007 to 31 March 2008. Data sources were routine records of RNTCP and NACP—specifically TB registers, TB treatment cards, ICTC registers, ART centre records, and the referral forms. Modified TB treatment cards were used to document the information related to referrals and HIV status. Trained field investigators visited all health facilities in the districts at least monthly for data collection and for updating the information and did not interfere in the referral procedure. Events abstracted from relevant records included registration of a patient in the TB register, initial ascertainment of HIV status, referral for HIV testing, HIV test results, referral to ART centres, evaluation for ART and initiation of ART as well as cotrimoxazole prophylaxis. Periodically the study investigators cross verified the data abstracted with that in the records available at the health centres, ICTC and ART centres.

The abstracted data on the line list was double entered into EPI info 6.04d (Centers for Disease Control and Prevention, Atlanta, USA) and entries in the data base were validated by randomly crosschecking with the abstracted data. Analysis was conducted using SPSS 10.0 (SPSS Inc., Chicago, USA). Proportions were compared by applying the χ^2^ test or Fisher's exact test if numbers were small.

### Ethics Statement

This evaluation assessed the feasibility and effectiveness of the implementation of new policies and procedures for referral and HIV testing of TB patients in the national programmes. Individual informed verbal consent was taken for all HIV testing as per NACP guidelines, with pre-test counselling, and post-test counselling by trained health care workers. Individual informed consent for data abstraction from routine programme records was not taken, as the collection of these data was considered by the institutional ethics committees and the National TB and HIV/AIDS Programmes to be a programme evaluation. Data abstracted from programme records were securely held and confidentially maintained by study staff. These procedures were specifically approved by the respective ethics committees of the National Tuberculosis Institute, Bangalore, and the Tuberculosis Research Centre, Chennai. Approval for the research was granted by the Ministry of Health and Family Welfare, Government of India.

## Results

### Referral and HIV Testing of TB Patients

**Figure 1:**
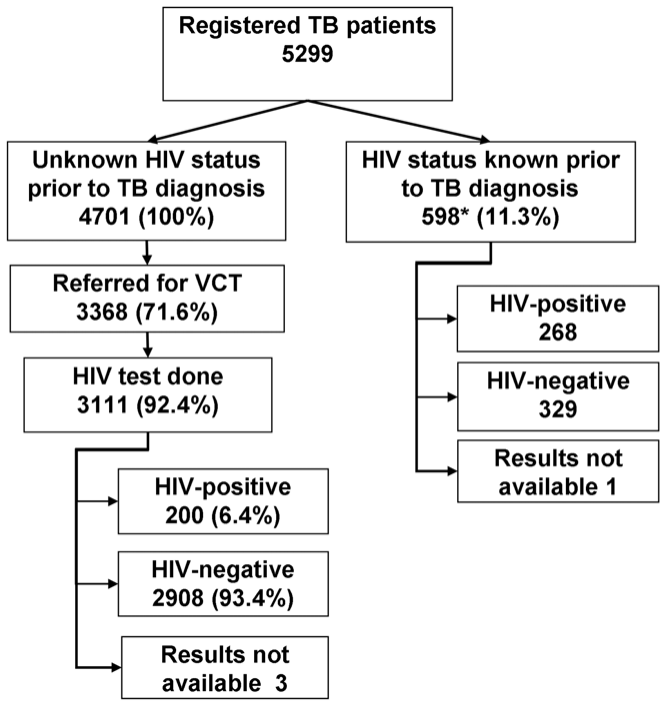
HIV referral and testing outcomes of RNTCP-registered TB patients, Mysore and Tiruchirappalli Districts, July 2007–March 2008.

From July 2007 to March 2008, 5299 TB patients were registered (**Figure 1**). Of these, 597 (11.3%) knew their HIV status at the time of TB diagnosis. Among the remaining 4,701 TB patients with unknown HIV status, 3,368 (71.6%) were referred for HIV testing. Among those referred for HIV testing, the median interval from the starting of TB treatment to referral for testing was 8 days (25%–75% interquartile range 0–28 days). Within 2 weeks of starting TB treatment, 62% of TB patients with initially unknown HIV status were referred for HIV testing. Of the 3,368 persons referred to the ICTC for HIV testing, 3,111 (92.4%) were actually tested. There were no significant differences in the age or sex distribution between those TB patients with unknown HIV status and those who were HIV tested (data not shown).

In total, HIV status was ascertained for 3,705 (70%) of the 5,299 registered TB patients. There was no difference in the ascertainment of HIV status between male or female TB patients (71% vs. 69%, p = 0.08), or between patients in Mysore or Tiruchirappalli districts (69.5% and 70% respectively) (**Table 1**). The proportion of persons with HIV status ascertained was lower in the young (<14 years) and elderly (>55 years) age groups, relative to the 15–55 age group (64% vs. 72%, p<0.01); however, within each age group, no significant differences were observed between males and females.

**Table 1:** Diagnosis of HIV among TB patients, Mysore and Tiruchirappalli, July 2007–March 2008.

Category	Subcategory	Registered N = 5299	HIV status ascertained (%)*	HIV-positive (%)*
District of Residence	Mysore	2,796	1,946 (69.5)	193 (6.9)
	Tiruchirappalli	2,503	1,763 (70.4)	275 (10.9)
Males	Total	3,464†	2,452‡(71)	344 (9.9)
	0–14 years	196	113 (58)	10 (5.1)
	15–24 years	429	311 (73)	7 (1.6)
	25–34 years	596	439 (74)	98 (16.4)
	35–44 years	763	552 (72)	150 (19.7)
	45–54 years	657	476 (72)	52 (7.9)
	55–64 years	502	360 (72)	19 (3.8)
	≥65 years	319	200 (63)	7 (2.2)
Females	Total	1,835	1,257 (69)	124 (6.8)
	0–14 years	147	76 (52)	3 (2.0)
	15–24 years	508	363 (72)	6 (1.2)
	25–34 years	410	294 (72)	47 (11.5)
	35–44 years	326	235 (72)	44 (13.5)
	45–54 years	226	156 (69)	18 (8.0)
	55–64 years	149	91 (61)	6 (4.0)
	≥65 years	69	42 (61)	0

* Denominator for percentage is the number of registered TB patients in each subcategory.

† Age for 2 patients not available.

‡ Age for 1 patient not available.

### HIV Prevalence among TB Patients

The overall prevalence of HIV infection among the 5299 registered TB patients was 468 (8.8%). This includes 268 (5.1%) who were known HIV-positive prior to TB diagnosis, and an additional 200 (3.8%) detected after TB diagnosis. Male TB patients were more likely to be HIV-infected than females (9.9% vs. 6.8%, relative risk [RR] 1.5, 95% confidence interval [CI] 1.2–1.8). Although HIV infection was detected in all age groups, HIV seroprevalence was highest in the 35–44 year age group both among males (19.7%) and females (13.5%) (**Table 1**).

### Characteristics of HIV-Infected TB Patients

Of the 468 HIV-infected patients, 268 (57%) had HIV diagnostic dates reported before TB diagnostic dates (**Table 2**). Among the 200 patients diagnosed with HIV on the day of or after their TB treatment initiation, the median time from TB treatment initiation to HIV diagnosis was 12 days (interquartile range 0–18 days). Among the 468 patients, 296 (63%) had pulmonary TB, and of these 107 (36%) were sputum smear-positive for acid-fast bacilli.

**Table 2:** Characteristics of HIV-infected TB patients, Mysore and Tiruchirappalli, July 2007–March 2008, N = 468.

Category	Subcategory	Number (%)
Type of TB (n = 468)	Pulmonary	296 (63)
	Extra pulmonary	172 (37)
Type of Pulmonary TB (n = 296)	New smear-positive	107 (36)
	New smear-negative	153 (52)
	Retreatment smear-positive	28 (9)
	Retreatment smear-negative	8(3)
Pulmonary smear status (n = 296)	Total smear-positive	135 (46)
	Total smear-negative	161 (54)
Time of HIV diagnosis (n = 468)	Prior to TB diagnosis	268 (57)
	After TB diagnosis	200 (43)
Time to HIV diagnosis (n = 200*)	Mean	12 days
	Interquartile Range (25–75%)	0–18 days
CD4 cell count (n = 468)	CD4 count <200/mm3	99 (21)
	CD4 count 201–350/mm3	24 (5)
	CD4 count >350/mm3	23 (5)
	CD4 count not available	146 (31)
	Not referred / not reached ART	176(38)
CPT provision (n = 468)	Yes	64 (14)
	No information	404 (86)
ART initiation (n = 468)	Before TB diagnosis	72 (15)
	After TB diagnosis	61 (13)
	No ART documented	335 (72)

* Among those persons with HIV diagnosis after TB diagnosis.

### Provision of Anti-Retroviral Treatment

**Figure 2:**
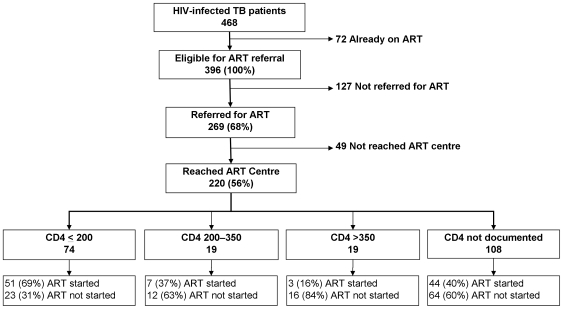
Referral of HIV-infected TB patients to ART centres, and initiation of ART during TB treatment by CD4 cell count, Mysore and Tiruchirappalli Districts, July 2007–March 2008.

Of the 468 HIV-infected TB patients, 72 (15.4%) were already on ART prior to TB diagnosis. Among the remaining 396 patients not yet on ART at the time of TB diagnosis, we documented that 269 (68%) were referred and 220 (56%) reached the ART centre (**Figure 2**). Of those who reached the ART centre, 105 (48%) started ART during TB treatment. In total, 177 (37%) out of 468 HIV-infected patients were documented to have received any ART during TB treatment. Cotrimoxazole prophylactic treatment provision was documented in 64 (15%) of the 468 patients.

CD4 cell count information was available for 112 (51%) of the 220 who reached the ART centre (**Figure 2**). Among the 112 with CD4 results available, 93 (83%) had a CD4 count <350/mm^3^ and were eligible for ART under NACO guidelines. Among the 74 patients documented to have a CD4 cell count of <200/mm^3^, ART was initiated in 51 (69%). Of the 19 with a CD4 cell count 201–350/mm^3^, ART was initiated in 7 (37%). The non availability of CD4 count information for 108 patients was unexpected and unexplained, as usually CD4 testing is conducted upon initial pre- registration at ART centres.

### Case Notification Before and After Initiation of PITC

**Figure 3:**
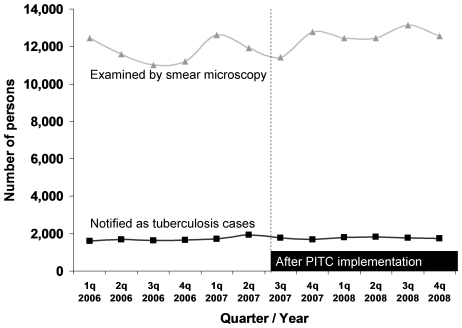
Number of persons examined by sputum smear microscopy, and number of notified tuberculosis cases, Mysore and Tiruchirappalli District, January 2006–December 2008.

Total TB case notification per 100,000 population for the period before and after the initiation of PITC is shown in **Figure 3**. No changes were noted in TB case-finding indicators after PITC implementation. The mean number of persons examined by smear microscopy was not different. The mean quarterly total TB case notification rate for 1.5 years prior to PITC implementation (January 2006 to June 2007) was 32 per 100,000 population; after PITC implementation (July 2007–March 2008), total TB case notification was 33 per 100,000 population.

## Discussion

This is the first study to provide tangible evidence from India of successful and timely implementation of PITC in TB patients through general health services in settings with high HIV prevalence. It also underscores the need for strengthening the uptake of ART services in such settings. In two districts, where HIV testing services were decentralized to a similar extent as TB services, half of TB patients with initially unknown HIV status were referred for HIV testing within 8 days of TB treatment initiation. This suggests that referrals for HIV testing were efficiently conducted, as would be necessary for patients to benefit from early HIV detection and treatment. After PITC policy implementation, HIV status was ascertained for 70% of TB patients, including 3108 TB patients (59%) who had previously unknown HIV status; 200 (3.8%) of TB patients were newly-detected as HIV infected. HIV status was ascertained most efficiently among adults aged 15–54 years, and with similar efficiency between males and females. Effective implementation of PITC for TB patients has been reported from other countries, including Thailand, South Africa and Kenya [12], [13], [14]. Earlier studies from India and Ethiopia warned of a possible gap between initial willingness for HIV screening and actual attendance at ICTC for testing [8], [15]. In this cohort, however, we observed that 92% of those referred for testing actually underwent HIV testing. We furthermore observed no effect of PITC implementation on TB case finding indicators, suggesting that apprehension of adverse effects on TB programme performance was unfounded.

Although HIV testing was reasonably efficient, the HIV status of 30% of TB patients still remained unknown, primarily attributable to lack of referral or incomplete documentation. Interviews of randomly selected patients from the same cohort, however, found that 80% of TB patients reported referral for HIV testing (submitted for publication manuscript No. 09-PONE-RA-13100). The probable reason for the discrepancy could be the gap in documentation of referral-related data on the programme records. These findings call for increasing the awareness among patients as well as health care providers about the high burden of HIV in TB patients and the advantages of early detection of HIV in conjunction with care and support services. Nursing and ancillary paramedical staffs at health facilities often have frequent contact and excellent rapport with patients, and are essential for maintaining accurate medical records. Greater involvement of ancillary health staff in referrals and documentation of results may enhance the efficiency of referrals as well as improve the quality of recording.

Although the two districts studied were considered to have high HIV prevalence on the basis of historical antenatal HIV surveillance results, the prevalence of HIV among adult TB patients was still somewhat surprising. HIV prevalence among TB patients was 8.8% overall, but among male TB patients aged 25–44 years, it was 18% or, more than 1 in 6 were HIV-infected. Male TB patients were 1.4 times more likely to be HIV-infected than female TB patients, consistent with the sex difference reported in national surveillance and the distribution of HIV in the general population [16]. While HIV infection among TB patients can occur at any age and was observed in all age groups in this population, the particularly high HIV seroprevalence among adult TB patients could be stressed in the message given by the provider to promote HIV testing.

The potential benefits of efficient detection of HIV infection among TB patients were unfortunately not effectively realized in these settings; of the 396 HIV-infected TB patients who were not already on ART, only 105 (27%) received any ART. The failure to efficiently provide ART to HIV-infected TB patients has also been reported from Thailand, Kenya and Malawi [17], [18], [19]. Even among TB patients with a documented CD4 count of <350/mm^3^ and eligible for ART as per NACP guidelines, only 62% actually received ART. We speculate that the provision of ART from a single center situated in a tertiary care setting in each of the two districts may be an important factor contributing to the limited ART uptake we observed. Operational research to understand weaknesses in the ART referral, evaluation, and initiation process at NACP ART centres is urgently needed.

### Limitations

As a programme evaluation this study was subject to some limitations. There were no control areas for comparison for the proportion of HIV-tested TB patients, as our intent was to establish the feasibility and effectiveness of PITC implementation under programme conditions across all general health facilities in both districts. We chose to contrast our observations relative to the larger target of HIV-testing for *all* TB patients in high HIV prevalence areas [7], [20]. We were not able to collect information on reasons for non-referral or failure to complete referrals for HIV testing, but a parallel study with patient interviews to address these questions from the patients' perspective has been conducted (submitted for publication, manuscript number 09-PONE-RA-13100). The rates of referrals, completion of HIV testing, and ART initiation, we report may underestimate what actually happened to these patients, as we depended on the ongoing process of recording the activities by providers which was updated till November 2008. We sought, however, to minimize the effect of non-recording by collecting information on each process from multiple data sources.

As the study was done under programme conditions, we only captured information on HIV testing done at NACP ICTCs and treatment provided from NACP ART centre, though HIV testing and ART is also available from the private sector. As per policy, however, persons with HIV test results from private laboratories were regardless referred to ICTCs for HIV counselling and testing on the premise that patients would benefit from counselling as well as confirmation of HIV status with quality-assured tests. Our information on CPT was limited to prescription from the NACP ART centre. We had limited information on CD4 results, due to recording challenges at the ART centre or patients not reporting back to ART centers due to multiple visits required for evaluation. Most patients who reached the ART centre would have a CD4 test done, hence—with half the CD4 information missing—our results regarding ART initiation across different levels of immune deficiency should be interpreted with caution. We could not assess the effect of bias due to under-reporting.

### Translating Research Findings into Policy and Practice

These results were considered by the National Programmes to be reasonably applicable to settings with higher HIV burdens, particularly since PITC was implemented by existing health staff with routine programme recording and reporting. We did provide additional training support as an external input, but training and supervision would normally be the role of the national Programmes. Also, HIV diagnostic care and treatment services under NACP are undergoing extensive expansion and decentralization, which would further improve opportunities for HIV diagnosis for TB patients and subsequent linkage to ART.

Based on these encouraging findings, PITC for TB patients has been incorporated into policy and practice in higher HIV burden settings in India [21]. To improve efficiency of HIV testing and the delivery of HIV treatment to HIV-infected TB patients, training on TB-HIV activities is being conducted for medical and paramedical staff throughout the general health system, with special emphasis on states with the highest HIV burden. Routine TB programme records have been altered to allow the recording of HIV status and links to care. RNTCP has begun routinely monitoring and publicly reporting on the proportion of TB patients with HIV status ascertained, the proportion HIV-infected, and the proportion of HIV-infected TB patients provided with ART and CPT [22].

### Conclusion

The implementation of PITC for TB patients is feasible in the general health care system of high HIV prevalence settings in India, and can efficiently identify HIV-infected TB patients. Concerns that PITC for TB patients might adversely affect TB case notification appear to have been unfounded. Our observation, however, of low ART uptake strongly underscores the importance of expanding and improving the delivery of treatment services for HIV-infected patients. Creating and enhancing awareness among providers and patients regarding the benefits of early diagnosis of HIV, along with benefits of care and support is essential.
